# Positive Selection and the Evolution of *izumo* Genes in Mammals

**DOI:** 10.1155/2012/958164

**Published:** 2012-08-21

**Authors:** Phil Grayson, Alberto Civetta

**Affiliations:** Department of Biology, University of Winnipeg, 515 Portage Avenue, Winnipeg, MB, Canada R3B 2E9

## Abstract

Most genes linked to male reproductive function have been known to evolve rapidly among species and to show signatures of positive selection. Different male species-specific reproductive strategies have been proposed to underlie positive selection, such as sperm competitive advantage and control over females postmating physiology. However, an underexplored aspect potentially affecting male reproductive gene evolution in mammals is the effect of gene duplications. Here we analyze the molecular evolution of members of the *izumo* gene family in mammals, a family of four genes mostly expressed in the sperm with known and potential roles in sperm-egg fusion. We confirm a previously reported bout of selection for *izumo1* and establish that the bout of selection is restricted to the diversification of species of the superorder Laurasiatheria. None of the *izumo* genes showed evidence of positive selection in Glires (Rodentia and Lagomorpha), and in the case of the non-testes-specific *izumo4*, rapid evolution was driven by relaxed selection. We detected evidence of positive selection for *izumo3* among Primates. Interestingly, positively selected sites include several serine residues suggesting modifications in protein function and/or localization among Primates. Our results suggest that positive selection is driven by aspects related to species-specific adaptations to fertilization rather than sexual selection.

## 1. Introduction

Molecular evolutionary analysis of protein coding genes show that most genes evolve under purifying selection with few exceptions of rapid change between species driven by positive selection [1, 2]. One class of rapidly evolving genes are those linked to reproduction, and in many cases positive selection has been found to drive their evolution towards species-specific adaptations. Broad-sense sexual selection [3] can lead to rapid evolution of primary sexual traits and genes that function largely to improve reproductive success, thus sexual selection has been suggested as an explanation for the rapid adaptive diversification of reproductive proteins. In mammals, many male reproductive genes have shown evidence of rapid evolution driven by positive selection [4–6]. It has been more difficult, however, to link positive selection to sexual selection, and numerous hypotheses have been proposed to explain this difficulty [7, 8]. 

One possible explanation is that most genes in mammals are members of gene families that have experienced several rounds of gene duplication. After duplication, genes can follow different paths in terms of their evolutionary trajectory such as the loss or adoption of new functions in different branches within a phylogeny [9]. Also, the use of phylogenetic-based methods to test for selection might be affected by localized bouts of positive selection to specific phylogeny branches. For example, two semenogelin genes transferred in the male's ejaculate contribute to the formation of a copulatory plug in promiscuous species, but only SEMG2 has shown evidence of positive selection that associates with differences among proxies of sexual selection in both Rodents and Primates [10–12]. SEMG1 has shown no evidence of selection using phylogenetic-based tests, but a population genetics study has shown evidence of a selective sweep with low polymorphism in chimpanzees and high divergence driven by positive selection between humans and chimpanzees [13]. Moreover, gorillas, with a polygamous mating system, have lost SEMG1 gene function entirely [14]. These studies seem to suggest that sexual selection bouts have been phylogenetically restricted or perhaps “softened” for SEMG1 but not SEMG2. 

The ADAM gene family contains a number of testes and sperm expressed genes of significant reproductive importance in mammals. Glassey and Civetta [15] found that codon sites within the adhesion domains (regions often linked to cell-cell adhesion) of ADAM2 and ADAM32 in mammals were under positive selection driven by male/female interactions at the molecular level. Subsequent work by Finn and Civetta [16] associated positive selection at ADAM 2 and ADAM18 to the level of promiscuity in primate mating systems (a well-documented proxy of sexual selection). They also noted that the signal of positive selection seen at ADAM18 disappeared when the phylogeny was expanded to include mammals other than primates. This lends further support to the hypothesis that bouts of sexual selection are often restricted to specific phylogenetic groups [16].

The izumo sperm-egg fusion gene (*izumo1*) is a member of the immunoglobulin superfamily (IgSF) of proteins with an important role in sperm-egg fusion. Gene knockouts of *izumo1 *produce sterile males whose sperm is unable to fuse to the egg membrane of zona pellucida free eggs [17]. Three other testes-expressed *izumo* genes (*izumo2*, *izumo3, *and *izumo4*) have been recently identified in mammals, with only *izumo4* being expressed in testes and nonreproductive tissues [18]. Although no formal tests of orthology and paralogy have been conducted, sequence comparisons across all *izumo* genes between human, mouse, rat, bull, and dog with the exception of *izumo3* (human, mouse, rat, and guinea pig) have shown that all four *izumo* genes have eight conserved cysteine residues within 144 amino acids with four *α*-helices hypothesized to exist between these residues [18]. This gene region has been named the izumo domain.

Here we first test orthology among *izumo* genes in mammals. We then explore whether positive selection is widespread across *izumo* genes in mammals or clade-specific, suggesting possible protein subfunctionalization. Our results show that *izumo* genes duplicated before the diversification of mammals and most likely prior to the diversification of vertebrates. Interestingly, most *izumo* genes show no evidence of positive selection and in one case clear relaxation of selective constraints (*izumo4* in Glires). The detected signals of positive selection within genes are either driven by ancient mammalian bouts of selection (*izumo1*) or more recent mammalian group diversification (*izumo3*) with no clear evidence to suggest any role for sexual selection during the diversification of this gene family in mammals.

## 2. Materials and Methods

### 2.1. Sequence Data Collection and Phylogenetic Reconstruction

We retrieved nucleotide and amino acid sequences from 23 (*izumo1*), 21 (*izumo2*), 13 (*izumo3*), and 20 (*izumo4*) mammalian species from Ensembl (see Supplementary Table  1 available online at doi:10.1155/2012/958164 for accession numbers). Sequences for all four genes were aligned using the global alignment algorithm ClustalW and visually inspected [19]. To maximize the number of informative sites during phylogenetic reconstructions, we limited the number of species included in the analyses to 16 for *izumo1* and 13 for *izumo2*, *izumo3*, and *izumo4*. To confirm that gene sequences labeled as members of the same *izumo* gene each formed a single monophyletic clade, the entire gene family phylogeny was reconstructed. Different phylogenetic tree models were tested based on protein alignments using the ProtTest 2.4 Server [20]. We choose the model with the lowest AIC (akaike information criterion) value as it takes into account both the likelihood of the model and the number of parameters contained within a model. Phylogenies for the individual *izumo* genes were reconstructed following the same protocol. Phylogenetic reconstructions were carried out using Maximum Likelihood in Mega 5.05 and the reliability of the tree branching assessed using 1,000 replicate bootstraps [21, 22].

### 2.2. Tests of Selection

Tests of selection were performed using codeml within Phylogenetic Analysis by Maximum Likelihood (PAML; v 4.4e) [23, 24]. For each *izumo* gene, the likelihoods of the M7 and M8 models were calculated to identify phylogenetic groups that had experienced positive selection. The M7 model assumes that *ω* values lie between 0 and 1 across the sequences, which is indicative of purifying selection or neutral evolution. The M8 model allows *ω* to exceed 1, which is characteristic of positive selection. To ensure that relaxed selection was not misinterpreted as positive selection, the likelihood of the null model M8a (where *ω* is fixed at 1) was also compared to that of M8 [5, 25]. When codons within an alignment were identified as being under positive selection, the Bayes Empirical Bayes (BEB) method was utilized to identify the specific codon sites under positive selection [26]. Codon sites with a posterior probability of positive selection higher than 90% were further evaluated based on the nature of the amino acid substitution occurring between species (e.g., cysteine residues influencing secondary structure and phosphorylation sites that can change the function or localization of a protein). We also mapped positively selected sites within protein domains described in the literature [18] or identified using the Motif Scan server [http://myhits.isb-sib.ch/cgi-bin/motif_scan], GenBank's protein site descriptions [http://www.ncbi.nlm.nih.gov/], and through the protein summary page on Ensembl [http://uswest.ensembl.org/index.html].

## 3. Results

### 3.1. Phylogenetic Relationship among *izumo* Gene Family Members

**Figure 1:**
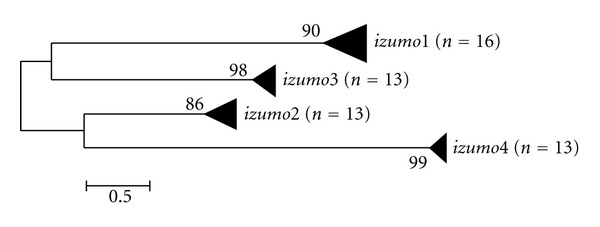
Molecular phylogeny of the *izumo* gene family emphasizing the formation of independent clades. *n* is the number of species included in each gene clade. The evolutionary history was inferred by using Maximum Likelihood and the JTT+G+F substitution model. The tree is drawn to scale, with branch lengths measured as the number of amino acid substitutions per site. The reliability of the tree branching was assessed using 1,000 replicate bootstraps. Bootstrap values lower than 50 are not shown.

The JTT model of sequence evolution [27] with G, the shape parameter for the gamma distribution, and F, the amino acid frequencies, was identified as the best fit for phylogenetic reconstruction of the *izumo* gene family according to its AIC value (JTT+G+F: lnL = −14693.74; AIC = 29641.5). Results were similar when using a JTT+I+G+F model, where I is the proportion of invariant sites (Table 1). The phylogeny supports orthology of all mammalian *izum*o genes (Figure 1). The duplication events that gave rise to gene family members are ancestral to the diversification of mammals and most likely vertebrates as predicted *izumo* genes from zebrafish, anole lizard, and the living fossil coelacanth (Genbank: XP_002663229, Ensembl: ENSACAP00000018570, and Ensembl: ENSLACT00000025764, resp.) were orthologs to their mammalian counterparts instead of forming outgroups (data not shown).

**Figure 2:**
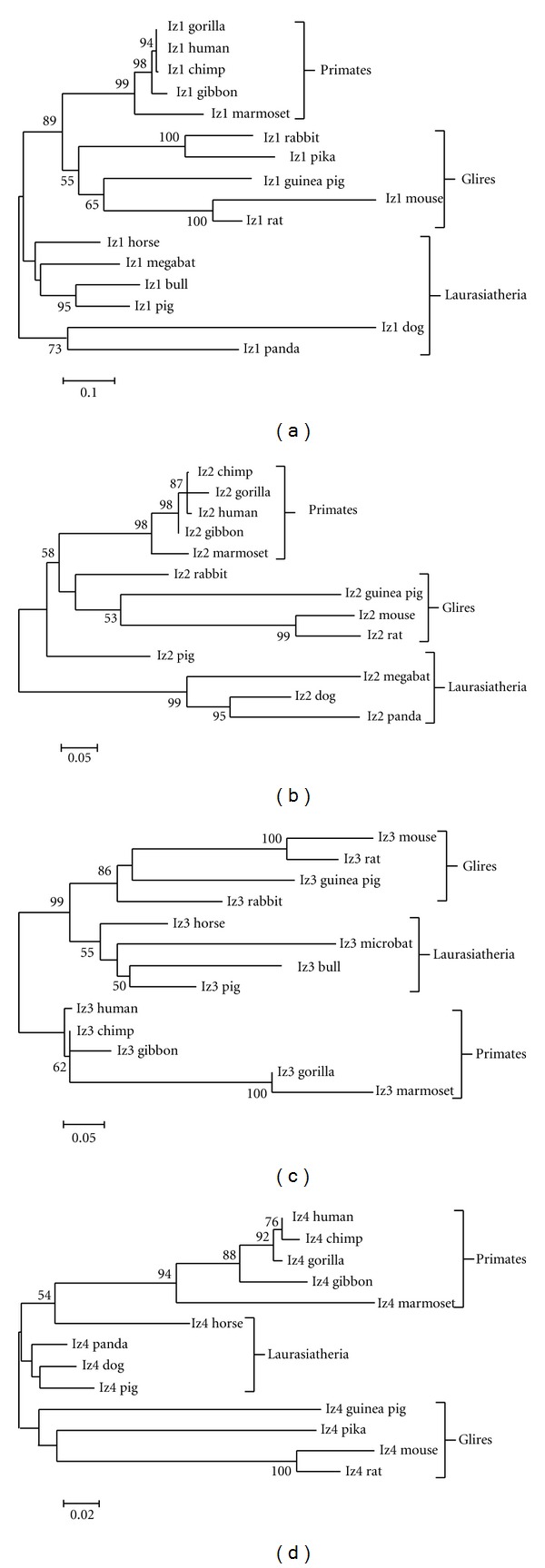
*Izumo* genes phylogenies. Molecular phylogenetic relationships of mammalian species within* izumo1* (a), *izumo2* (b), *izumo3* (c), and *izumo4* (d) emphasizing the formation of common clades between orthologs (superorder Laurasiatheria, clade (grandorder) Glires, and order Primates). The evolutionary history was inferred by using Maximum Likelihood and different JTT substitution models (see Table 1). The trees are drawn to scale, with branch lengths measured as the number of amino acid substitutions per site. The reliability of the tree branching was assessed using 1,000 replicate bootstraps. Bootstrap values lower than 50 are not shown.

We then tested organismal clades within each *izumo* gene phylogeny. ProtTest identified JTT+I+G+F as the best model for *izumo1* and *izumo2* (lnL = −6533.3; AIC = 13166.7 and lnL = −3022.1; AIC = 6132.2, resp.). Models JTT+G and JTT+I+G were found as the best fits for *izumo3* and *izumo4* (lnL = −2710.3; AIC= 5468.6 and lnL = −2302.7; AIC = 4655.4, resp.) (Table 1). All *izumo* genes show good support for Primates as a distinct clade (bootstrap values higher than 90%) but grouping within the Glires, which includes orders Lagomorpha and Rodentia, and within the superorder Laurasiatheria were weaker. The best bootstrap values for Glires (86%) and Laurasiatheria (89%) as distinct clades were for *izumo3* and *izumo1, *respectively (Figure 2). Particularly poor was the resolution of Glires and Laurasiatheria as two separate clades for *izumo4* (Figure 2). 

### 3.2. Evidence of Selection for *izumo* Genes

A previous study had identified evidence of positive selection at *izumo1* using M8 *versus *M8a models within PAML in a phylogeny including human, rat, and mouse, plus a combination of either chimpanzee or macaque and dog or bull [10]. We replicated the analysis and confirmed previous results, but noted that the detection of positive selection was sensitive to the species included in the analysis as we failed to detect positive selection when the tree included both bull and chimpanzee (2Δ*ℓ*_M8a-M8_ = 2.48; *P* = 0.12). This PAML analysis was subsequently carried out for *izumo2*–*4 *with all four possible species combinations for each gene. Positive selection was only identified once more: when dog, human, macaque, mouse, and rat were examined together for *izumo2* (2Δ*ℓ*_M8a-M8_ = 10.29; *P* = 0.001).

Tests of selection were subsequently performed with additional species that were found to cluster into the groups of Primates, Glires, or Laurasiatheria for each gene. Different combinations of species were utilized to include both the maximum number of sequences available per group, as well as the same species across all four genes. Among Primates, *izumo2* (2Δ*ℓ*_M8a-M8_ = 6.10; *P* = 0.014) and *izumo3* (2Δ*ℓ*_M8a-M8_ = 8.91; *P* = 0.003) showed evidence of positive selection but the signal of selection for *izumo2* was dependent on the species included in the analysis (Table 2). None of the *izumo* genes showed evidence of positive selection among Glires. *Izumo1* appeared more highly conserved than others based on comparisons of models' likelihoods, with *izumo4* showing evidence of relaxation of selective pressures (Table 2). Finally, we only found evidence of positive selection for *izumo1* among Laurasiatheria, and the results were consistent regardless of the species included in the analysis (2Δ*ℓ*_M8a-M8_ = 30.22; *P* < 0.001 and 2Δ*ℓ*_M8a-M8_ = 5.92; *P* = 0.015, resp.) (Table 2).

**Figure 3:**
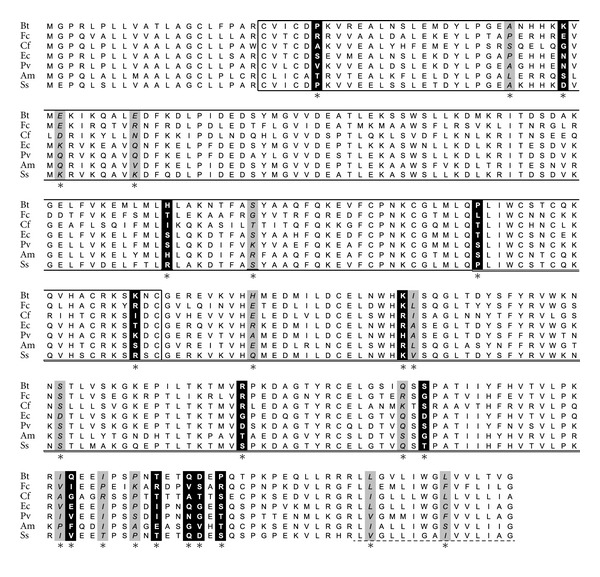
Amino acid sequence alignment of IZUMO1 for species of the superorder Laurasiatheria. Distribution of positively selected sites (see asterisks) with posterior probability higher than 90% (grey background/italic font) and 95% (black background/white font). The izumo domain is boxed (single black line), the immunoglobulin domain is double underlined, and the transmembrane domain is underlined by a dotted line. Two letters are used to indicate species: Bull/Cow (*Bos taurus*—Bt), Cat (*Felis catus*—Fc), Dog (*Canis lupus familiaris*—Cf), Horse (*Equus caballus*—Ec), Megabat (*Pteropus vampyrus*—Pv), Panda (*Ailuropoda melanoleuca*—Am), and Pig (*Sus scrofa*—Ss).

**Figure 4:**
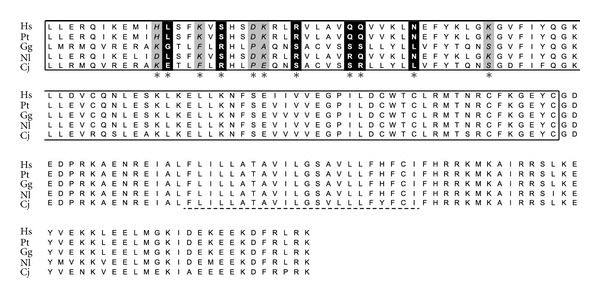
Amino acid sequence alignment of IZUMO3 for species of the order Primates. Positively selected sites and protein domains are indicated as in Figure 3. Two letters are used to indicate species: Human (*Homo sapiens*—Hs), Chimpanzee (*Pan troglodytes*—Pt), Gorilla (*Gorilla gorilla*—Gg), Gibbon (*Nomascus leucogenys*—Nl), and Marmoset (*Callithrix jacchus*—Cj).

Among *izumo1 *sequences for species of the superorder Laurasiatheria, nine amino acid sites within the izumo domain (representing 6.4% of the domain), nine within the immunoglobulin domain (8.5%), and ten at the caboxi-end of the protein (21%), two of which fell within the 15 site transmembrane domain (13%), had posterior probabilities higher than 90% of being under positive selection (Figure 3). Among Primates, only two amino acids within *izumo2* had posterior probabilities higher than 90% of being under positive selection, with the two sites located within the izumo domain (data not shown) and the signal of selection being sensitive to the number of species included in the analysis. An estimate of *ω* per branch within the *izumo2* phylogeny showed that only when nine species of Primates were included, the branch leading to macaques had an *ω* higher than one (**ω** = 1.31). All eleven positively selected sites at *izumo3* among Primates were located within the first half of the izumo domain (11%) (Figure 4). Five of the eleven amino acid sites, and four out of six with posterior probabilities higher than 95% of being under positive selection, included substitutions involving serine residues, suggesting modifications in protein function and/or localization. It is interesting to notice a pattern of preservation of the serine residues only between human, chimpanzee and gibbon, or gorilla and marmoset (Figure 4).

## 4. Discussion

 We have not found a consistent pattern of selection for a specific *izumo* gene across phylogenetic groups but rather signals that are gene and phylogenetic group specific. This is evident by the fact that two *izumo* genes have experienced positive selection among species of different well-supported phylogenetic groups. Positive selection at *izumo1* in mammals appears to be driven by selective bouts that have primarily affected the evolution of the wider group of species within the Laurasiatheria superorder. Species included in the analysis for which we detected positive selection diversified on average approximately 80 MyA with the closest relatives being pig and bull (60.5 MyA) [28]. Thus, positive selection is most likely the consequence of an ancient bout of selection and not linked to species-specific adaptations. On the other hand, *izumo3* showed evidence of selection among more recently diversified species of Primates with the most diverged pairs of New World monkey and Apes diversifying approximately 42.5 MyA [28]. While the bout of selection at *izumo3* is more likely linked to species-specific adaptations, the fact that positively selected sites include a large proportion of serine residues that group marmosets and gorillas away from human, chimpanzee and gibbon suggests that the selective bout is not linked to sexual selection. This is because both groups, although small in numbers, include species with very different mating systems such as gibbons (monogamous) and chimpanzee (polygynandrous) and gorilla (polygynous) and marmoset (polyandrous).

It is unclear what drives the different patterns of positive selection at *izumo1* and *izumo3*. It has been recently shown that the IZUMO1 protein forms homo-multimers that are likely essential for the formation of a sperm membrane multiprotein complex with a crucial role in fertilization [18]. IZUMO1 has also been shown to form complexes that are reproductively nonessential with another protein (ACE3) in mice [29]. Ellerman et al. [18] have found evidence suggesting that the transmembrane domain and/or the cytoplasmic tail of IZUMO1 functions in forming multimers while the izumo domain is required for the formation of homo-dimers. Little is known about the function of *izumo3* but its expression is independent of the presence of *izumo1,* and the IZUMO3 protein appears to form only homo-dimers [18]. Thus, it is not surprising that IZUMO1, with its central role in mammalian fertilization and its unique ability to form multimers, would be more conserved among closely-related species groups, whereas *izumo3*, with a more limited number of protein interactions, could have evolved towards different adaptations among more closely related species.

The differences in the locations of the positively selected sites between *izumo1 *and *izumo3* are also interesting. The IZUMO1 protein has positively selected sites throughout an uncharacterized region and the izumo, immunoglobulin, and transmembrane domains. The transmembrane domain of IZUMO1 has been linked to the formation of multiprotein complexes, which have not been reported for any other IZUMO protein [18]. The IZUMO3 protein is only capable of forming homo-dimers, and all positively selected sites fell within the izumo domain, with no positively selected sites within the transmembrane domain or uncharacterized regions. This finding leads to two competing hypotheses. The ability to form both dimers and multimers is an ancient adaptively important function of *izumo* genes but all current members (except IZUMO1) have lost the ability to form multimers, or the ancestral *izumo* gene was only able to form dimers, and IZUMO1 gained the ability to form multimers sometime after the ancestral gene duplicated. The fact that positively selected sites within the transmembrane domain were only found in IZUMO1 for a group comparison that included the most distantly related mammalian species (Laurasiatheria), but no other species groups, lends indirect support to the idea that the multimer forming ability of *izumo1* is an ancient function. Thus, it is more likely that other *izumo* genes have lost this function. Moreover, nonmammalian orthologs have been found for *izumo1* in zebrafish and coelacanth and for *izumo2* in lizard. The zebrafish and coelacanth lineage is approximately 100 million years more ancient than the lizard lineage when comparing both to mammals [28], lending further indirect support to the hypothesis that the ability to form multimers is an ancestral state, although we must concede that this finding could be influenced by the current state of genomes functional annotations. 

Given that all four *izumo* genes are expressed in sperm, the different patterns of selection among mammalian groups lends support to protein subfunctionalization rather than neofunctionalization or gene loss, but the expression of *izumo4* in tissues other than the testes and sperm should not be overlooked. The rapid evolution found to be driven by relaxed selection in the Glires might be indicative of an ongoing process of gene loss [30]. Species-specific functional assays should be carried out on these genes and proteins to determine how an ancestral function might have been partitioned among gene family members (subfunctionalization) or whether new functions might have arisen from a simpler ancestral protein (neofunctionalization). 

Most *izumo* genes appear to have evolved among mammals under different levels of purifying selection. The fact that the divergence of several *izumo* genes cannot be linked to positive selection should serve as a cautionary message against a preconception that rapid divergence among testes-specific genes lends support to species-specific adaptations. Moreover, signals of positive selection at testes or sperm specific genes should not be directly associated with sexual selection as in many cases, particularly in mammals, such signals might be driven by clade or species diversification in fertilization requirements, immune-related challenges, or even influenced by patterns of genome duplication and the gene's genomic environment [7, 8].

## Figures and Tables

**Table 1:** Phylogenetic model testing for amino acid sequence alignments. Only the top three fits are shown.

Genes	Models	AIC	lnL
All	JTT+G+F	29641.5	−14693.7
	JTT+I+G+F	29642.4	−14693.2
	JTT+G	29647.6	−14715.8

*izumo1*	JTT+I+G+F	13166.7	−6533.3
	JTT+G+F	13168.7	−6535.3
	JTT+I+G	13173.5	−6555.7

*izumo2*	JTT+I+G+F	6132.2	−3022.1
	JTT+G+F	6133.8	−3023.9
	JTT+I+F	6147.6	−3030.8

*izumo3*	JTT+G	5468.6	−2710.3
	JTT+I+G	5470.6	−2710.3
	JTT+G+F	5471.6	−2692.8

*izumo4*	JTT+I+G	4655.4	−2302.7
	JTT+G	4657.4	−2304.7
	HIVb+I+G	4663.7	−2306.9

**Table 2:** Evidence of positive selection at *izumo* genes within three major groups of mammals.

Group	Gene	*n*	*p*_seq_	2Δ*ℓ* (M8-M7)	*p*_1_; *ω*	2Δ*ℓ* (M8-M8a)
Primates	*izumo1*	9; 5	0.66; 0.99	1.94; 0.62		
	*izumo2*	9; 5	0.72; 0.81	6.79^∗^; 0.15	0.04; 5.39	6.10^∗^
	*izumo3*	5	0.73	9.03^∗^	0.36; 2.36	8.91^∗∗^
	*izumo4*	9; 5	0.73; 0.76	4.19; 3.43		

Glires	*izumo1*	7; 4	0.46; 0.86	1.33; 1.17		
	*izumo2*	6; 4	0.83; 0.99	4.33; 3.64		
	*izumo3*	4	0.91	4.82		
	*izumo4*	5; 4	0.76; 0.83	10.99^∗∗^; 4.57	0.08; 1.48	0.94

Laurasiatheria	*izumo1*	7; 4	0.77; 0.82	39.75^∗∗∗^; 7.03^∗^	0.24; 2.57	30.22^∗∗∗^; 5.92^∗^
	*izumo2*	6; 4	0.48; 0.76	0.31; 0.88		
	*izumo3*	4	0.68	4.85		
	*izumo4*	6; 4	0.66; 0.79	0.00; 0.00		

*n*: number of species included; *p*_seq_: proportion of sequence analyzed after removing gaps; 2Δ*ℓ*: the difference in likelihood estimates from different models of selection (see methods); *p*_1_: proportion of codon sites under positive selection; *ω*: d_ N_/d_S_ per codon for sites under positive selection. ^∗^ *P* < 0.05; ^∗∗^ *P* < 0.01; ^∗∗∗^ *P* < 0.001.
